# Marine Biodiversity in the Australian Region

**DOI:** 10.1371/journal.pone.0011831

**Published:** 2010-08-02

**Authors:** Alan J. Butler, Tony Rees, Pam Beesley, Nicholas J. Bax

**Affiliations:** 1 CSIRO Wealth from Oceans National Research Flagship, Hobart, Tasmania, Australia; 2 CSIRO Marine and Atmospheric Research, Hobart, Tasmania, Australia; 3 Australian Biological Resources Study, Canberra, Australian Capital Territory, Australia; 4 CSIRO Marine and Atmospheric Research, Hobart, Tasmania, Australia; Institut Pluridisciplinaire Hubert Curien, France

## Abstract

The entire Australian marine jurisdictional area, including offshore and sub-Antarctic islands, is considered in this paper. Most records, however, come from the Exclusive Economic Zone (EEZ) around the continent of Australia itself. The counts of species have been obtained from four primary databases (the Australian Faunal Directory, Codes for Australian Aquatic Biota, Online Zoological Collections of Australian Museums, and the Australian node of the Ocean Biogeographic Information System), but even these are an underestimate of described species. In addition, some partially completed databases for particular taxonomic groups, and specialized databases (for introduced and threatened species) have been used. Experts also provided estimates of the number of known species not yet in the major databases. For only some groups could we obtain an (expert opinion) estimate of undiscovered species. The databases provide patchy information about endemism, levels of threat, and introductions. We conclude that there are about 33,000 marine species (mainly animals) in the major databases, of which 130 are introduced, 58 listed as threatened and an unknown percentage endemic. An estimated 17,000 more named species are either known from the Australian EEZ but not in the present databases, or potentially occur there. It is crudely estimated that there may be as many as 250,000 species (known and yet to be discovered) in the Australian EEZ. For 17 higher taxa, there is sufficient detail for subdivision by Large Marine Domains, for comparison with other National and Regional Implementation Committees of the Census of Marine Life. Taxonomic expertise in Australia is unevenly distributed across taxa, and declining. Comments are given briefly on biodiversity management measures in Australia, including but not limited to marine protected areas.

## Introduction

### The region

We consider here the Australian marine jurisdictional area from shore to the boundary of the Exclusive Economic Zone (EEZ). This comprises mainland Australia, Tasmania, and offshore islands including sub-Antarctic islands, but not the Australian Antarctic Territory, which is covered by the Antarctic regional synthesis [1]. Within this region, we consider all ecosystems – coastal, continental shelf, offshore, ranging from tropical to sub-Antarctic.

**Figure 1:**
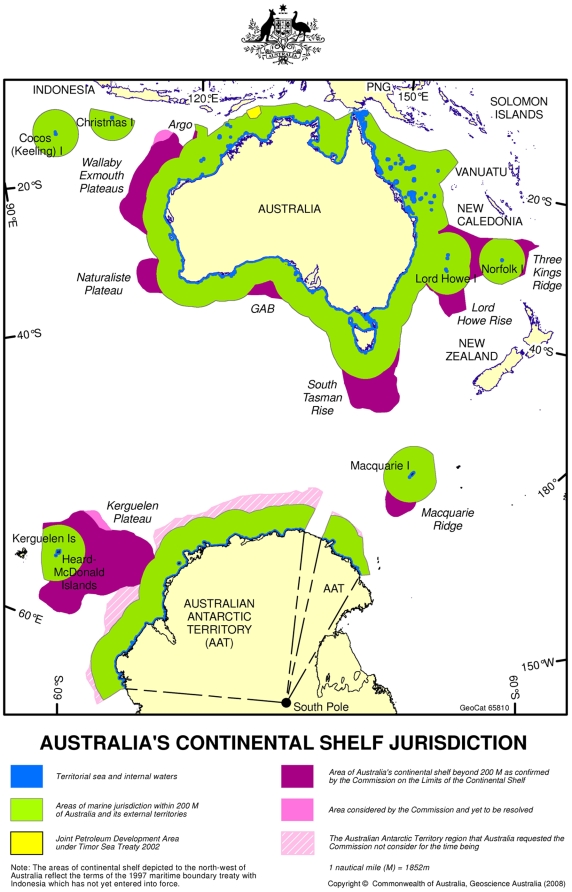
The boundaries of Australia's Exclusive Economic Zone and Continental Shelf Jurisdiction. This paper concerns this whole area with the exception of the Australian Antarctic Territory, which is covered by the Census of Antarctic Marine Life synthesis [1].

The Australian EEZ including the Territorial Sea (Figure 1) is about 9.0 million km^2^ in area, with a further 2.04 million square km in Australia's Antarctic Territory, and an additional confirmed Extended Continental Shelf of 2.56 million km^2^. The mainland EEZ has a coastline of about 36,000 km, and spans more than 5,000 km from the tropics (9° S) to temperate latitudes (47° S). Even considering only shallow water, this extensive continuous coastline, together with about 12,000 islands from the tropics to the polar region, contains a wide range of tropical to sub-Antarctic shallow water conditions and habitats. Together with the deepwater areas of the continental shelf and the slope, the deeper abyssal regions, and the overlying water column, this constitutes a vast array of highly diverse habitats and ocean features; many have received limited, if any, exploration and we can only touch on a few aspects here.

### Physical, geological, chemical, oceanographic, and biological structure of the region

Condie and Harris [2] describe interactions between physical, chemical, biological, and sedimentological processes in Australia's shelf seas and provide an excellent synopsis of earlier studies of these processes and of the geological, oceanographic and biological structure of the region. Australia was shaped by its rifting away from Gondwana (the part that is now Antarctica) beginning in the Cretaceous (estimates vary from ∼125 to ∼83 Ma), the formation of the Tasman Sea in the late Cretaceous to Eocene, and eventual collision, 10–15 million years ago, with the Pacific Plate. It is now bounded by three oceans (Pacific, Southern, and Indian) and four marginal seas (Timor, Arafura, Coral, and Tasman). In addition, this chapter considers the parts of the Australian EEZ that are in sub-Antarctic waters (Macquarie Island, a remarkable modern upthrust from a mid-oceanic ridge in the Southern Ocean, and Heard Island and the MacDonald Islands on the Kerguelen Plateau) as well as offshore islands near the continent (Christmas and Cocos-Keeling Islands in the Indian Ocean and Lord Howe and Norfolk Islands in the Pacific). The regional current systems are important; the Leeuwin Current on the west coast is unique as a poleward-flowing eastern boundary current, and has an important influence on the ecosystems of the west coast. The East Australian Current is a normal, poleward-flowing western boundary current, again with a strong influence on the ecosystems of that coast. Both major boundary currents are influenced by basin-scale ocean-atmosphere processes (e.g., the El Niño-Southern Oscillation and the Indian Ocean Dipole) and are likely to be strongly influenced by climate change (the signals can be seen already), with significant consequences for the biota [3]. Australia's shelf seas are considered oligotrophic; the continent is mostly arid and there are no major coastal upwellings (cf. the Benguela system). There is much more that could be said and, as Condie and Harris [2] show, the Australian region is heterogeneous; this means that for purposes of management, and for our purposes here in describing the biota, it is necessary to subdivide it into reasonably natural “bioregions.” This has been done at several scales, using available geological, physical oceanographic, and biological information.

### Biogeographic subdivisions

A biogeographic analysis, or “bioregionalization,” of nearshore waters was undertaken in 1998 by the Interim Marine and Coastal Regionalisation for Australia (IMCRA) Technical Group [4]. The available data were limited and methods used for the IMCRA process differed somewhat between states. In 2005, the National Oceans Office commissioned the National Marine Bioregionalisation of Australia (NMB) for waters beyond the shelf [5]. Data on bathymetry, demersal fish, sponges and sediments, and oceanographic data, were used to identify a suite of unique seafloor bioregions comprising 41 provinces, three depth-related biomes on the continental slope, and geomorphic units that represent clusters of geomorphic features around the EEZ. Physical properties of the water and satellite estimates of primary productivity were used separately to describe 25 different water masses in Australia's oceans, identified by different circulation regimes and oceanographic features.

IMCRA Version 3.3 and the NMB have been combined to create IMCRA Version 4.0 [6], where the ‘I’ now stands for “Integrated.” IMCRA 4.0 identifies provinces, mesoscale regions, and geomorphic units.

IMCRA will continue to be refined. Recently, the range information on short-ranging demersal fish species on the continental shelf (which had not been included in the NMB) has been examined by Lyne et al. [7] as a project within the Commonwealth Environment Research Facilities (CERF) Australia's Marine Biodiversity Hub (http://www.marinehub.org/). Thus, a refinement of the regionalization is now available, including depth-related biomes on the continental shelf. At about the same time, O'Hara [8], [9] prepared a bioregionalization based on brittle stars (Ophiuroidea), which can now be compared with the findings for fish. A survey off Western Australia found that the bioregionalization based on fish was coincident with patterns in six sampled invertebrate phyla [10]. The majority of available biodiversity data have not been collected with sufficient spatial resolution to be referred to the finer bioregions in IMCRA 4.0, but the Australian Faunal Directory (AFD) database (see below) is indexed by IMCRA 4.0 provincial bioregions. IMCRA 4.0 also classifies its 24 provinces and 17 transition zones according to whether they are tropical, subtropical, warm-temperate, or cold-temperate. For this paper, we have extracted data, where possible, according to IMCRA 4.0 provinces and transition zones (adding one called HIMI for Heard Island and the MacDonald Islands). These highly subdivided data contained many zeros and are not presented in this paper although the authors have them available for later use.

For waters beyond the continental shelf, a subdivision of the Australian EEZ into 13 Large Marine Domains (LMDs) was developed by the Division of Marine Research of the Commonwealth Scientific and Industrial Research Organisation (CSIRO) [11] and these have been used in support of regional marine planning under Australia's Oceans Policy [12]. These LMDs were used in Large Marine Ecosystems of the World 2002 [13] and by Spalding *et al*. [14] as the province level in their hierarchical scheme of Marine Ecoregions of the World. We note that in Australia, for biogeographic and planning purposes, the LMDs have largely been superseded by the NMB and now by IMCRA 4.0, but it will be convenient for the present paper to group our data into the LMDs for comparison with other Census of Marine Life regions. Accordingly, IMCRA 4.0 provincial bioregions have been grouped to an equivalent LMD.

### History of research and species discovery

Australia has been occupied by people for some 40,000 to 60,000 years. The Aboriginal people accumulated much knowledge of its flora, fauna, and ecological systems, including those of its “sea country,” but much of this knowledge and understanding remains cryptic. European scientific study began with the first scientifically staffed voyages of discovery, notably those of James Cook in 1770 with Joseph Banks and Daniel Solander aboard (who collected almost nothing marine!) [15], Nicolas Baudin in 1801-3 with François Péron aboard [16], [17], and Matthew Flinders in 1802 with Robert Brown and Ferdinand Bauer aboard [18]. Charles Darwin visited Australia in the *Beagle* in 1836 [19]. The voyage of HMS *Challenger*, 1872-76 included Australian samples in its global investigation of the deep sea, and its reports are a basis of many disciplines. After the establishment of the colony of New South Wales by the British in 1788, it was not long before the colonies (later to become the states of Australia) established various scientific societies and natural history museums, which were very active, and published the results of their scientific endeavors in a variety of journals as proceedings, transactions, and records (e.g., [20]). Discovery in the sea was of course more difficult and more limited than on land, but there was much activity during the twentieth century; by mid-century, substantial biogeographic syntheses and ecological interpretations were possible; see, e.g., [21].

Considerable effort was made on the taxonomy and descriptive ecology of organisms on accessible shores: some examples include [22], [23] and more recently [24]. This has developed into a strong tradition of experimental ecology on seashores and in shallow water (see, e.g., [25]) as well as a determined effort to produce identification guides (see Text S1).

Publications on phytoplankton have been available in Australia since the 1930s (tabulated by regions in [26]), but species lists are available only for limited locations. The treatment of phytoplankton in the Ocean Biogeographic Information System (OBIS) is weak compared with other groups. There is even less understanding of microorganisms and only limited and patchy knowledge of zooplankton; research on zooplankton ecology has increased recently, but is not yet finding its way into OBIS. Although Australian waters are considered oligotrophic (and are generally clear and low in planktonic abundance) there are, nevertheless, seasonal blooms with locally high productivity and rapid turnover in the plankton in at least some places in Australia (e.g., [27], [28]). The study of plankton, and of benthopelagic coupling, is less well developed than the study of benthos in Australia.

Beginning in the first half of the twentieth century, there was energetic research targeted at fisheries by Australian state agencies and by CSIRO Division of Fisheries and its predecessors [29]. Although searching for commercial prospects, this work collected many noncommercial fish and invertebrates that were lodged in museums throughout the country, including the Australian National Fish Collection at CSIRO Marine and Atmospheric Research (CMAR). These fish collections have recently provided the most comprehensive and useful biological dataset for bioregionalization of Australian waters. In the 1960s, a period of intensive environmental research began, targeting in particular bays, estuaries, and shelf near major capital cities and initiating a new and energetic period of environmental and taxonomic research [30]–[39].

More recent work has explored deeper waters, with interests in exploration, the conservation of biodiversity and research on sustainable fisheries [10], [40]–[48], [76]. Thus, museums of Australia and the world are building significant collections of Australian specimens from depths as great as 2,000 m and, in restricted parts of the shelf and slope, quite comprehensive faunal collections (see below).

### Census of Marine Life activities within Australia

The National Committee (chaired initially by Ian Cresswell, now by Nicholas Bax) comprises Australian representatives from Census of Marine Life projects – Census of Antarctic Marine Life (CAML), Census of Coral Reef Ecosystems (CReefs), REEFS, History of Marine Animal Populations (HMAP), Pacific Ocean Shelf Tracking Project (POST), Global Census of Marine Life on Seamounts (CenSeam), Continental Margin Ecosystems on a Worldwide Scale (COMARGE), and Tagging of Pacific Predators (TOPP) – and is also linked to the Barcode of Life initiative. OBIS Australia (http://www.obis.org.au) provides data on Australian taxa to OBIS; at present some 30 percent of Australian taxa are represented on the OBIS database (more detail below). The Great Barrier Reef Seabed Biodiversity Project is a Census-affiliated project. These projects will report their findings separately from this article. The Census has links to many institutions and organizations at national and state levels, including museums, academia, and policy groups.

The Commonwealth Environment Research Facilities (CERF) Program, a new Australian Government initiative supporting public good research, funds Australia's Marine Biodiversity Hub (Director, Nicholas Bax; http://www.marinehub.org/index.php/site/home/). The Hub, building on historic data collections, old and recent surveys conducted by Hub partners, state agencies, national and international fisheries groups, provides national leadership in describing, predicting, and managing Australia's marine biodiversity, and works closely with Census projects. Its work is particularly pertinent to the questions mentioned below in the Discussion section, regarding the use of surrogates, both to interpolate given existing data and to design future surveys.

## Methods

### Potential data sources

Biological samples collected from Australian waters by numerous workers over a long period are held in many repositories. We do not attempt here to analyze individual collections, but mostly concentrate on retrieving biological information from several readily accessible electronic databases. However, we mention other sources here:

1. *General collections held by museums and similar institutions.* A number of institutions in Australia collaborated to develop the Online Zoological Collections of Australian Museums (OZCAM), an online distributed query network to faunal collections in Australian museums (http://www.ozcam.gov.au/search.php). Participants (although not all of them contribute marine data to OZCAM) are: Australian Biological Resources Study, Australian Museum, CSIRO, Museum and Art Gallery of the Northern Territory, Museum Victoria, Queensland Museum, Queen Victoria Museum and Art Gallery, South Australian Museum, Tasmanian Museum and Art Gallery, and Western Australian Museum. OZCAM aims to link the databases of the museums of Australia so that they can be queried simultaneously through a single portal. A subset of the current OZCAM is linked to OBIS, but by no means all museum records are currently digitized and therefore available through OZCAM. Here, we merely note that extensive marine collections (many faunal and floral groups) are held by Australia's museums and herbaria; the subset represented by OZCAM was analyzed for this study.

2. *Faunal groups interpretable over a limited area.* For a small number of taxonomic groups some limited synthesis can be presented. These groups include algae, some mollusks, polychaetes, ascidians, vertebrates, and some crustaceans. They have had consistent sampling over much of the Australian EEZ and extensive taxonomic study by a currently active scientific group.

3. *Groups analyzed biogeographically on a national scale.* There are a small number of groups for which the above is true and, *in addition*, a biogeographic analysis of the data has recently been attempted. These include fish, brittle stars, decapod crustaceans, and sponges.

The National Marine Bioregionalisation of Australia [5] included a benthic bioregionalization based on bathymetry, data on demersal fish, sponges and sediments, and oceanographic data. Thus, demersal fishes and sponges have been analyzed on a large biogeographic scale, concentrating on depths beyond the slope.

The data on demersal fish [49] were used as surrogates for the rest of the marine biota, as they were considered at the time to be the only available biological dataset with adequate national spatial coverage and taxonomic resolution to provide robust analysis of broadscale biogeographic patterns. Data on the spatial distribution (latitude and longitude) and depth distribution of 1,489 demersal fish species from 494 genera (representing 121 families) were collated by CSIRO Marine Research for use in the benthic bioregionalization. Data for this project originated from a variety of sources, including research surveys, fisheries catches, museum collection records, and literature records. The form used for the bioregionalization is essentially the limits of records; the assumed ranges of species are obtained through interpolation. Thus, the bioregionalization dataset may tend to overestimate the occurrence of species, especially close to the range limits. Conversely, the point data in OBIS will usually underestimate occurrence and often range limits.

The dataset on sponges [50], though identified by the working group as the only one (apart from fish) of value at that time for bioregionalization, is more limited than the fish dataset. It includes data from collections held at the Queensland Museum, Australian Institute of Marine Science, Museum and Art Gallery of the Northern Territory, and Western Australian Museum. It is limited to sponges found in the tropical waters of Australia's EEZ, ranging from Brisbane in the east to North West Cape in the west. The sponge dataset consists of point data, including genus and species names, latitude and longitude, and water depth. The resulting database contains about 3,800 species (where a species is defined as a distinct operational taxonomic unit) from more than 4,000 localities and represents a total of 425 genera, 120 families, 26 orders and 3 classes, of which about 2,250 species occur in marine waters of tropical Australia. The interpretation [50] contains a great deal of analysis on a scale too fine for this paper.

Reports on the National Marine Bioregionalisation are accessible online through http://www.environment.gov.au/coasts/mbp/imcra/nmb.html and data and products through http://www.environment.gov.au/erin/dig/index.html. These include the National Marine Bioregionalisation GIS, individual project reports and associated figures, fact sheets, and large-format plot files. The National Marine Bioregionalisation resulted in the production of a number of marine datasets, and these datasets are maintained and updated by the respective custodians. Datasets and custodians associated with the National Marine Bioregionalisation are as follows:

- Australian Bathymetry Database (Geoscience Australia)
- National Sediments Database (MARS) (Geoscience Australia)
- Demersal Fish database (CSIRO Marine and Atmospheric Research)
- National Sponge database (Queensland Museum, through the Online Zoological Collections of Australian Museums)
- CSIRO Atlas of Regional Seas (CARS)/Oceanographic database (CSIRO Marine and Atmospheric Research)

More recently, the 1,489 fish species with distributional records in the demersal fish database have been increased fivefold and the distributions of short-ranging demersal fish species on the shelf have been analyzed [7], as have brittle stars [8], [9]. National collaboration to standardize taxonomic descriptions of sponges will greatly expand the range of the National Sponge Database. National biogeographic maps of polychaetes are being developed. Selected fish, brittle stars, and certain crustaceans, are now the subject of genomic studies in Australia's Marine Biodiversity Hub, which will lead to refined phylogenetic and phylogeographic interpretation.

4. *Detailed surveys.* Within the Australian EEZ, certain localities have been sampled thoroughly using consistent methods for a wide range of taxa and depths, producing detailed datasets. These include the Great Barrier Reef World Heritage Area, and some nearshore, outer shelf and upper slope locations, such as Bass Strait, Northwest Shelf, and Dampier Archipelago. Considerable attention has been paid to refining methods of survey, making use of physical data to refine the design of biological sampling. Within the Census, this “surrogate-based” approach is being addressed in the Census synthesis project Predicting Seabed Species and Environments. Also many fine-scale surveys have been undertaken in shallow nearshore (diver-depth) and intertidal waters by numerous state agencies and university groups.

Australia's Marine Biodiversity Hub is interested in surrogacy and methods for prediction of biodiversity given limited data. To facilitate that work, it has audited available biological datasets from various sources that provide broad spatial scale, extend over a wide range of contrast in surrogates, and have broad coverage of taxa. These are generally presence/absence/abundance datasets from surveys with representative sampling. Metadata has been obtained for the most suitable available datasets, and the majority of them have been acquired for the Hub's use. Many are also in the metadatabase MarLIN (http://www.cmar.csiro.au/marlin/) maintained by CSIRO Marine and Atmospheric Research (CMAR). The progress report of this Data Audit and Acquisition Project is available. It lists 56 metadata entries, which include references to cruise reports and publications. About eight of the datasets have unrestricted access, and 31 others would be available by consultation with the researchers or custodians. Some of these entries refer to massive survey efforts that recorded large numbers of species. To illustrate, here is part of one entry, concerning only one component of the data from the Great Barrier Reef Seabed Biodiversity Project (GBR SBBP):

*Title: Seabed Biodiversity on the Continental Shelf of the Great Barrier Reef World Heritage Area (Epibenthic Sled)*

*MarLIN record number: 7036 Anzlic Identifier: ANZCW0306007036*

*Abstract: The benthic invertebrate, plant and fish biodiversity of the 200,000 km^2^ area of the GBR shelf seabed was sampled by a 200 m tow of a 1.5 m epibenthic sled at 1191 sites, representing a full range of known physical environments, during six 1-month-long voyages on the AIMS [Australian Institute of Marine Science] vessel Lady Basten. More than 7,000 species/species-equivalent OTUs (operational taxonomic units) were identified. The dataset comprises 79,173 site-by-species records. …etc.*

Data from some other surveys (e.g., in Bass Strait) are in OZCAM. There is, in principle, much that could be done with these datasets from the viewpoint of the Census, but it cannot be prepared for this paper. There are, however, still gaps in many areas, and very little sampling beyond 1,000–2,000 m in depth.

5. Other data exist in published records (literature), unpublished database records, and nondigital survey records, such as field data sheets.

6. Regional checklists and summaries of flora and fauna: there are a number of published lists, and databases on restricted taxonomic groups; most are not used for this paper.

7. An important source of information that is not an electronic database is the report *Numbers of Living Species in Australia and the World* (2006, 2009) [51], [52], prepared by Arthur Chapman for the Australian Biological Resources Study (ABRS), a Program of the Department of the Environment, Water, Heritage and the Arts. This is largely a synthesis of published information and information supplied by researchers from around the world. This publication considers whole taxonomic groups and does not distinguish marine from nonmarine. The 2009 edition is available online at http://www.environment.gov.au/biodiversity/abrs/publications/other/species-numbers/index.html.

### Datasets used for this paper

Because of time constraints, data for analysis were restricted to the following electronic databases:

- OBIS. There are potentially about 50,000 recognized marine species in the Australian region (see Table S1), of an estimated 230,000 globally [53]. OBIS contains data for records with a latitude/longitude entry, and probably includes some 25–30% of Australian taxa (some 13,000 species in the Australian region in the wider sense, that is, the EEZ and adjoining seas). The OBIS Australia Web site is located at www.obis.org.au. Data of some 10,000 records have been supplied by Australian institutions and organizations including CSIRO, OZCAM (data from the museums of Australia), other Australian museums with records not in OZCAM, the Australian Antarctic Division, the Australian Institute of Marine Science (AIMS) and the Bureau of Rural Sciences. Also Australian data of some 3,000 records were made available from international sources – surveys done by researchers from other nations, some collation exercises (e.g., FishBase), and overseas museum holdings. OBIS can be searched by a latitude/longitude bounding box, 10 degrees square or smaller, by named EEZ, certain predefined Large Marine Ecosystems, or by species or higher groups (e.g., mollusks, fishes, birds). OBIS has comprehensive datasets for some faunal groups (e.g., fish), but many groups have only partial datasets.
- CAAB. The Codes for Australian Aquatic Biota database maintained by CSIRO Marine and Atmospheric Research (www.cmar.csiro.au/caab) lists about 25,000 marine species, that is, about half of the estimated number of recognized Australian species, mainly with a single reference as an entry into the relevant literature (a citation that the species does occur in this region). Thus, CAAB is useful for a species-count in the Australian region, but not for finer-scale biogeographic questions. The main gaps in species listings are in phytoplankton, protists, and a few invertebrate groups. CAAB currently contains codes and taxonomic information for the following aquatic organisms in the Australian region:
  - Over 4,500 fish taxa, representing virtually all known marine and freshwater species in Australian waters;
  - Other marine vertebrates (reptiles, birds, and mammals), representing all currently recognized marine species in Australia;
  - Around 20,000 codes for marine invertebrates in Australian waters, including sponges, stony corals, echinoderms, commercially important crustaceans and mollusks, tunicates, and other taxa;
  - Codes for Australian seagrasses and mangroves, representing all currently recognized marine species;
  - A preliminary selection of Australian seaweeds and microalgae.
- AFD. The Australian Faunal Directory, maintained by the Australian Biological Resources Study (ABRS), lists some 15,000 taxa recognized as marine in this study, that is, about 30% of anticipated named Australian marine species, a subset of which carry broad scale information about distribution (by IMCRA bioregion – see below). AFD is freely available on the Web, but extensive data requests can be obtained directly from to ABRS. Since it is a regional checklist and not a source of individual georeferenced data points, the Austraflian Faunal Directory is *not* represented in OBIS. AFD contains statistical summaries for taxa and is available at http://www.environment.gov.au/biodiversity/abrs/online-resources/fauna/afd/home.
- OZCAM. As of August 2009, the Online Zoological Collections of Australian Museums database contains about 1.52 million data records for some 69,000 animal species, of which about 26,900 are considered to represent marine species in the Australian region (a significant proportion of these records are also available in OBIS). The OZCAM data are also exported to the Global Biodiversity Information Facility data portal, www.gbif.org, and are accessible by that route as well as through the OZCAM portal.
- The database NIMPIS (National Introduced Marine Pest Information System – part of the National System for the Prevention and Management of Marine Pest Incursions) [54] details introduced species in Australia and documents those that are potential invaders. However, it is only now being updated after a five-year hiatus. Annotations about the status of introduced species are available in AFD, but these records are incomplete. Here, the work of Sliwa et al. [55] has been used. It provides estimates of nonnative (known to have been transported to Australia, which is outside its native range, by human activities) and cryptogenic (those that cannot be confirmed as either native or nonnative) marine species in Australia from two sources: surveys of 41 ports around Australia, and an extensive literature review to identify nonnative species transported by shipping.

The data used for this article are all freely available. The ABRS, CAAB, OBIS and OZCAM data are all freely available from the respective websites. The corrected and deduplicated combination used for the totals in Table 1 and Table S1 is not currently in the public domain but is freely obtainable on request from author TR. Metadata for all other datasets are in the public domain.

**Table 1:** A summary of the number and knowledge of Australia's marine species by major taxa.

Taxonomic group	No. species^1^	State of knowledge^2^	No. introduced species^3^	No. experts^4^	No. ID guides^5^
**Domain Archaea**	0	2			
**Domain Bacteria (including Cyanobacteria)**	9	2			
**Domain Eukarya**					
**Kingdom Chromista**					
Phaeophyta	325	5	6		2
**Kingdom Plantae**				“algae” 8	
Chlorophyta	279	4	2		2
Rhodophyta	966	4	10		2
Angiospermae	75	5			5
**Kingdom Protoctista (Protozoa)**				3	1
Dinomastigota (Dinoflagellata)	286	4	2		
Foraminifera	120	3			
**Kingdom Animalia**					
Porifera	1,701	4	4	3	1
Cnidaria	1,754	4	10	5	4
Platyhelminthes	536	3	1	4	1
Bryozoa	1,062	4	22		1
Mollusca	8,525	5	20	13	7
Annelida	1,558	4	10	14	3
Crustacea	6,365	4	24	16	5
Echinodermata	1,594	5	3	4	2
Urochordata (Tunicata)	866	5	2	1	2
Other invertebrates	893	3		8	11
Vertebrata (Pisces)	5,184	5	12	18	18
Other vertebrates	265	5		11	5
**SUBTOTAL**	32,363				
**TOTAL REGIONAL DIVERSITY** 6	32,897				

1 Number of species in the databases CAAB, OBIS, AFD, and OZCAM.

2 State of knowledge: 5 =  very well known; 4 =  well known; 3 =  poorly known; 2 =  very poorly known; 1 =  unknown. For full definitions see Table S1.

3 Number of introduced species from [55].

4 Number of taxonomic experts from an ABRS survey in 2003; figures may include present and past practicing experts.

5 Identification guides cited in Text S1.

6 Total regional diversity including all taxonomic groups as reported in Table S1.

### Data extraction

The AFD was queried for taxa listed in Table S1 by IMCRA 4.0 provincial bioregions. The resultant data were provided to one of the authors (TR) and were integrated with data from CAAB, OBIS, and OZCAM. Although the databases have many species in common, each database does contain some species that are not found in the others. Data were further filtered according to occurrence in the present region of interest (by literature resources or supplied latitude and longitude coordinates), and by habitat type (to include known or likely marine or brackish organisms, and exclude those from other habitats), the latter by recourse to relevant marine and nonmarine flags in the IRMNG (Interim Register of Marine and Nonmarine Genera) database maintained at CMAR as a component of OBIS Australia (see www.obis.org.au/irmng/). Known synonyms (for example as referenced in the Catalogue of Life and Australian Faunal Directory) and variant spellings were also reconciled so far as was practicable to reduce multiple counting. The analysis considered described species only; unnamed species and taxa of uncertain attribution (e.g., “?*Halitiara* sp. 2”) were excluded.

Available AFD data were essentially complete (but were augmented a little with data from CAAB, OZCAM, and OBIS) for the following groups:

Phylum Porifera
Phylum Platyhelminthes (marine species)
Phylum Brachiopoda
Phylum Nematoda
Phylum Arthropoda, Subphylum Crustacea:
  Subclass Phyllocarida
  Subclass Eumalacostraca: Superorder Hoplocarida, Superorder Peracarida, Superorder Eucarida
Phylum Mollusca: Class Aplacophora, Class Polyplacophora, Class Scaphopoda, Class Cephalopoda, Class Gastropoda (in part)
Phylum Sipuncula
Phylum Echiura
Phylum Kamptozoa
Phylum Annelida: Class Polychaeta, Class Pogonophora, Superclass Clitellata (marine species)
Phylum Echinodermata
Phylum Hemichordata
Phylum Chordata:
  Subphylum Tunicata
  Subphylum Cephalochordata
  Subphylum Vertebrata: Higher Taxon Agnatha, Superclass Pisces, Classes Mammalia, Reptilia, Aves (marine species)

For the remaining groups, data came mostly from a combination of CAAB, OZCAM, and OBIS records.

The result was the best estimate of (1) the number of species for each higher taxon represented in AFD, CAAB, OZCAM, and OBIS combined, eliminating the majority of duplicates. From a range of sources (frequently [52]) we estimated Australian species either (2) recognized but not yet in AFD, CAAB, OZCAM, or OBIS, or (3) likely to occur in Australian waters, as a roughly estimated proportion of known global biodiversity, but not yet reported. The total of (1) + (2) + (3) gives us an estimate of the number of Australian species that are in some sense “anticipated known.”

Finally, where some expert comment had been made either to Chapman [52] or to us, we recorded an estimate of the number of species NOT in the categories above. These are either collected, but not yet even described as new taxa, or awaiting discovery. These estimates are of course very uncertain.

For faunal groups with sufficient point data, the counts for IMCRA 4.0 provincial bioregions were combined, according to Table S2, to correspond to Australia's Large Marine Domains, which constitute the provincial level in [14]. This gave meaningful results only for 17 higher taxa for which there were sufficient data. They are tabulated by LMD in Table S3.

Information about state of knowledge is based largely on expert opinion, but our starting criterion was the proportion, of those species estimated to occur in Australia, that are listed in the four major databases. For available taxonomic expertise, we used figures from the 2003/6 survey by the Australian Biological Resources Study (ABRS). ABRS will be conducting another survey in the near future. Data on “unknown” species were based on expert opinions. Data on introduced species are included in the AFD and were extracted by IMCRA 4.0 province, along with the general species counts, but that information is known to be incomplete. In Table S1, we have given figures from Sliwa et al. [55]. Data on threatened and otherwise listed species come from the database of the Department of Environment, Water, Heritage and the Arts (http://www.environment.gov.au/index.html), which is responsible for these listings under the *Environment Protection and Biodiversity Conservation Act 1999* (EPBC Act).

### Identification guides

Identification guides should be authoritative, and reasonably comprehensive for their particular taxonomic scope, and they should include illustrations and, preferably, keys. They should not simply be species lists, however thorough, on the one hand, nor be beautifully illustrated but incomplete field guides to common and conspicuous species, on the other. Many guides to the flora and fauna of Australia are available, arising from the activities of scientific institutions and societies across the country, the leadership of inspired individuals, and the activities of the Australian Biological Resources Study. Some, although important reference works, do not fit the definition above (examples are the volumes in the *Fauna of Australia* and the *Zoological Catalogue of Australia* produced by ABRS, see Bibliography 2 in Text S1). It seems that no comprehensive bibliography exists of identification guides for the whole of Australia, although Edgar's *Australian Marine Life* [24] gives a broad overview of available resources for at least the temperate (southern) portion of Australian waters. A partial search has been made for such works in the preparation of this paper – see Bibliography 1 in Text S1.

## Results

### Numbers of Australian marine species

Table S1 shows the following estimates:

1. The number of species represented in AFD, CAAB, OZCAM, and OBIS combined, eliminating the majority of duplicates. (Column headed *Total species in AFD+CAAB+OZCAM+OBIS*)

2. The total number of Australian species that are in some sense “anticipated known.” These are the above, plus estimates of numbers of species described but not yet included in AFD, CAAB, OZCAM, or OBIS, plus estimates of numbers of named species currently unrepresented in collections but considered likely to be detected in Australian waters given adequate sampling. (Column headed *Australian known marine species (est.)*)

3. The likely number of “unknown” Australian species. These are either collected but not yet described as new taxa, or new (unnamed) taxa awaiting discovery. Estimates of these are based only on expert opinion and only for a few groups, and are very uncertain. (Column headed *Australian unknown species (est.)*)

The findings are recorded in Table S1 and summarized for major groups of organisms in Table 1, where “No. of species” refers to the number of species in the four major databases (item 1 above), and “State of knowledge” reflects the difference between item 1 and item 2 above, as well as qualitative opinions about the available expertise and available identification guides. Described species listed in the three major databases number 32,897, as at October 2009, of which 30,303 are animals. Introduced species number 129, of which 108 are animals. It is estimated that there are about 50,000 “known” Australian marine species, of which almost 48,000 are animals. “Unknown,” and largely undiscovered, species are of course difficult to estimate. Table S1 shows expert-based estimates for some groups.

The available data are subject to a number of errors. The limitations of coverage of the databases are mentioned in the Methods section. It is the aim of the custodians of AFD, CAAB, and OBIS that all Australian described species will eventually be included (ABRS has a mandate to consider all Australian flora and fauna, terrestrial and marine; CAAB and OBIS are marine). However, all of the databases reflect their origins and histories, and each has characteristic gaps. Thus, this tabulation represents the best picture of marine species at the time of writing. In particular, there are a number of zero values, where currently we have no data. A few of these may be real zeros (for example, there are no marine bryophytes), but most reflect existing knowledge that has not yet been included in the databases, or groups where studies are not yet far advanced. A further potential error is that of multiple counting of taxa that are recorded under either slightly different name variants, or as unrecognized synonyms, the latter either synonymized taxa originally bearing different names (heterotypic synonyms) or the same taxon transferred into different genera by different authors (homotypic synonyms). The bulk of these duplicates have been removed by a sequence of algorithmic processing followed by manual inspection, but some undoubtedly remain, though probably at a low level (estimate under 1%–2% of names currently held).

### Species diversity by bioregion

The subdivision of the data by is further restricted because many records in the databases lack locational information (a species is simply known to occur in “Australia”). As a result, we have data for only 17 higher taxa (Table S3). They show a variety of patterns, and the North Eastern LMD appears to be the most species rich.

### Threatened species

Australia recognizes a range of levels of threat, and in addition to listing species as formally threatened (in other words, with some level of risk of extinction), it lists a number of others as protected. These various measures are explained at http://www.environment.gov.au/coasts/species/index.html.

The Australian Government lists threatened species under the *Environment Protection and Biodiversity Conservation Act 1999* (EPBC Act). A number of marine species (all vertebrates) are listed as critically endangered (2 fishes, 2 birds), endangered (3 fishes, 3 reptiles, 6 birds, 2 mammals), vulnerable (8 fishes, 2 reptiles, 21 birds, 6 mammals), and conservation dependent (3 commercially exploited fishes) (http://www.environment.gov.au/cgi-bin/sprat/public/publicthreatenedlist.pl?wanted=fauna). The Act also provides for the identification of threatened ecological communities. No marine communities have so far been listed, but two are being considered. In addition to identified threatened species and communities, a substantial number of others – all vertebrates – are “listed” under the Act (http://www.environment.gov.au/coasts/species/marine-species-list.html), which gives them protection against being killed, injured, or traded.

The states have comparable legislation, though generally less powerful than the EPBC Act. The state listings of species differ slightly from those under the EPBC Act, but not so as to change the above picture significantly. A number of marine species, all vertebrates, are listed as threatened and more are listed as protected, and in some states protected species include certain plants (e.g., mangroves and seagrasses).

Threatened species are also typically included in the Marine Bioregional Profile for each regional marine plan prepared under the EPBC Act. Profiles are now on the Department of the Environment, Water, Heritage and the Arts for the South Eastern, South Western, North Western, North, and East regions (visit http://www.environment.gov.au/coasts/index.html).

## Discussion

### Known

Australia has the results from extensive surveys, but still these surveys have covered only a small part of the EEZ. A number of major species databases, including AFD, CAAB, OBIS, and OZCAM, have been developed, but still there is a large amount of information that has not been captured and remains as records in collections, the literature, or a paper-based form.

Analyses of species richness and distribution patterns have been attempted for a few taxa, such as fish, certain echinoderms, decapods, and sponges in a limited part of the EEZ. On the other hand, there are comprehensive faunal collections for limited areas. This means that it is possible to compare the fauna of different biomes (e.g., [56]) and to ask questions about the relationships between faunal composition and physical and chemical variables, and thus to develop a surrogate-based approach both to the efficient design of future surveys and to interpolation within existing data. Australian researchers are active on both questions, developing new statistical approaches to analyze the data (e.g., [57]) and analyzing the data to determine whether fish, for example, are useful surrogates for other taxa (work by Australia's Marine Biodiversity Hub [10]).

Table 1 shows there are almost 33,000 marine species recorded in the four major databases. Most of these (over 30,000) are animals (the four databases are primarily faunal) with the balance being marine macrophytes, seaweeds, and some potential phytoplankton species. Some of the Australian algal groups are well known, but we are unable to assemble complete counts for this paper. It is also well known that some species have been recorded in the literature but not yet entered into the databases, and that many others have been either distinguished, but not yet formally described, or probably occur on account of their anticipated global distribution, but await adequate sampling to be reported. In Table S1, the column headed *Australian known marine species (est.)* represents estimates of their number and shows that there are about 50,000 anticipated known Australian marine species, of which almost 48,000 are animals. The difference between *Australian known marine species (est.)* and the *Total species in AFD+CAAB+OZCAM+OBIS* indicates the gap in our present electronic data holdings and survey effort to date, and is indicated in the “State of knowledge” column in Table 1.

Among the Australian fauna, vertebrates are a very well known group, although there is a continuing high rate of discovery of new fish and shark species. Invertebrates, in general, are less well known, but groups such as Porifera, Mollusca, Polychaeta, Crustacea (Malacostraca), and Echinodermata, which are supported by current taxonomic expertise, are among the better known of the marine animals. Other invertebrate groups remain poorly known with little, if any, taxonomic support or expertise (see Table 1).

Some of the faunal data contain sufficient information about location that interpretations regarding regional fauna are possible. This has been prepared for Australia's LMD [11], which are equivalent to the Large Marine Ecosystems in [13] and the provinces in [14], for the Australian region. The regional data are meaningful for 17 higher taxa (Table S3). They show a variety of patterns, and the North-east LMD (NE LMD) looks the most species rich. However, a number of caveats have to be discussed in interpreting these patterns (e.g., the NE LMD includes the well-studied Great Barrier Reef; certain taxonomists have concentrated on particular regions; and the datasets reflect the locations of recent surveys).

### Unknown

Clearly, kingdoms other than Animalia and Plantae are poorly represented in the databases used for this paper. Of them, some really are poorly known (Fungi, some Protoctista, and the domains Archaea and Bacteria, including Cyanobacteria), but there have been extensive studies of others at least for some parts of the Australian marine jurisdictional area (Rhodophyceae, Chlorophyceae, Phaeophyceae). It must also be recognized (Tables 1 and S1) that our information is poor for many groups in Animalia.

There has been only sparse sampling in most parts of Australia (exceptions are detailed above), even around the coastline. In principle this could be remedied, but in practice the resources required are very large. There have been few samples of any kind deeper than 2,000 m (examples include studies by Poore et al. [40], [58] and a recent *Jason* voyage to the Tasman Fracture Zone south of Tasmania) and there are few anywhere in Australia deeper than about 1,200 m. Early analysis of the recent *Jason* survey suggest distinct biomes or depth-delineated invertebrate communities deeper than 2,000 m, where the species and even the family may be new to science (R. Thresher, CSIRO, pers. comm.). Australia currently lacks the capability to explore the benthos in the deep parts of its EEZ (the deepest explorations, by the Autonomous Benthic Explorer *ABE* in 2008 and *Jason* in 2009, have both been by U.S. devices, although one was deployed from an Australian ship).

### Unknowable

The number of species remaining to be discovered is difficult to estimate. The column headed *Australian unknown species (est.)* in Table S1 represents such estimates. Even with well-known groups, new species are still discovered in well-studied areas (e.g., Pisces in the recent GBR SBBP); in less studied areas, a majority of species taken are new to science. Thus, in a cruise working in 100–1,000 m depths along the outer shelf and slope of Western Australia in 2005, the following numbers of species were collected (new species in parentheses):

- Sponges –108+ (new –70%)
- Soft corals –141 (118 new –80%)
- Mollusks –462 (310 new –67%)
- Echinoderms –326 (82 new –38%)
- Crustaceans –529 (167 new –30%)
- Ascidians –50+ (40 new –80%) [10]

Similarly, a cruise in two newly declared marine reserves south of Tasmania revealed the following:

- Sponges –469 (100% unknown)
- Cnidaria –102 (92% unknown)
- Mollusks –77 (74% unknown)
- Echinoderms –128 (60% unknown)
- Crustaceans –106 (75% unknown)
- Ascidians –19 (100% unknown) [76]

For Bass Strait shelf macroinvertebrates, 51% of 803 species were undescribed [41] and for Eastern Australian slope isopods, 90% of 359 species were undescribed [40].

It might thus be conservative to propose that only 20–30% of Australian species of these macrofaunal groups have been discovered, whereas the total for more cryptic groups, where we do not have current expertise, may be much higher. If we suppose as a starting figure for the whole biota that only 20% have been discovered, then the Australian marine biota is at least 250,000 species. This is undoubtedly an underestimate because surveys frequently sample only the larger species. In the above and other surveys, it is typical to catch half of the species only once (and this does not seem to change with the level of sampling effort employed to date). This implies that there are many more species out there that will never be sampled. However, it is not especially meaningful to speculate about the number of undiscovered species beyond noting that there are clearly many.

The Australian EEZ is enormous and Australia's seabed increased by 2.5 million km^2^ in 2008, when its claim was recognized under the UN Commission on the Limits of the Continental Shelf. We will, in the near future, make some surrogate-based inferences about likely diversity outside surveyed areas, but it is unimaginable that we could survey it in many lifetimes.

Although it can be estimated for some species (e.g., for sharks and rays), the number of endemic species is almost impossible to estimate overall, and we have not offered any figures. Estimating endemism for Australia should only be attempted by first categorizing our waters into tropical and temperate. Put simply, endemism is low in the tropical north where many species are shared with the Indo-West Pacific, whereas in the temperate south, endemism is relatively high at the species level. Figures up to 90% have been mentioned. For the well-understood decapods, it is 40% [59]. With low sampling density, endemism is difficult to establish. Even in an intense study like the GBR SBBP (above) many species are seen only once. This is insufficient to establish that they are restricted in distribution; we may simply be ineffective at sampling for them. In each shelf and slope cruise in Australia, many species collected are new to science and, again, seen once only; this could reflect the paucity of sampling, not their rarity. Even if comparable surveys had been done in similar habitats of marine regions to the north, east, and west (and they generally have not), this “sampling rarity” would make us cautious in drawing conclusions about endemism to the Australian region. Thus, with the sampling effort available now and in the immediate future, for the less intensely studied groups, endemism can be considered close to “unknowable.”

### Biases introduced

The Australian Biological Resources Study recently surveyed the resources and holdings of Australia's natural science collections [60]. Available expertise, of course, causes bias in the groups that can be used. Australia has been a leading nation (particularly considering its small population) in taxonomic research [53], but the loss of taxonomists in Australia is now a significant concern.

The ABRS *Survey of Australian Taxonomic Capacity (2003/2006)* [60] details the status of the taxonomic workforce and resources available to natural science collections. Further reckoning of the figures from this report determined that 43% of Australia's 149 taxonomists were more than 45 years old, 8% were over 60, and one-third of the taxonomic workforce was voluntary (ABRS, pers. comm.). Moreover, four full-time positions were being lost each year, while only one was gained, resulting in a net average loss of expertise at the level of three taxonomists annually. Recruitment of students to replace retiring specialists is low. ABRS predicted that in just five years, Australia will face a crisis in chronic lack of taxonomic capacity.

A National Taxonomy Forum was called by ABRS to discuss these matters in 2007. The forum also discussed strategies to overcome impediments to taxonomic research, and exciting new approaches, including DNA techniques and the development of the Atlas of Living Australia (http://www.ala.org.au/), an Australian Government-supported initiative. The critical need to digitize existing data (especially from museums), making it widely available for scientific research, was emphasized. Although development of tools to access existing collections may indirectly promote the need and support for fundamental taxonomic work in Australia, the scale of the decline in taxonomic expertise suggests that a more direct approach will be needed.

The ABRS Survey of Australian Taxonomic Capacity [60] asked what groups each researcher studied. They found that:

*The groups with the most researchers were the Dicotyledons (c. 40 people) then the insects (c. 25 people). No researchers were recorded as working on the Cephalochordates, Hemichordates, Monotremes, or Other Minor Acoelomates.*

**Figure 2:**
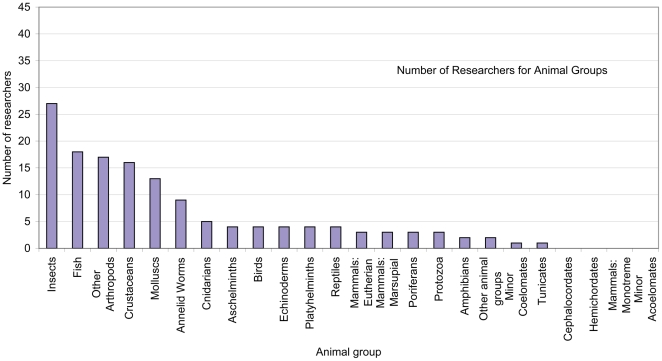
Number of researchers for animal groups, taken unaltered from the report of an Australian Biological Resources Study survey in 2003.

The results for animal groups are summarized in Figure 2.

Another aspect of *capacity* is the availability of research vessels; Australia's research fleet is limited, but will shortly increase substantially. As noted above, Australia currently has no capacity to explore the seabed below about 2,000 m, and no deepwater autonomous underwater vehicles (AUVs) or remotely operated vehicles (ROVs). Australia has a number of small (less than 30 m) research vessels, mostly operated by state agencies and some by the Australian Institute of Marine Sciences, and it has a specialized Antarctic supply vessel, the *Aurora Australis*, which carries out oceanographic and biological research in the Southern Ocean, in addition to its supply role. At present, Australia has only one other oceangoing research vessel, the *Southern Surveyor*, operated by CSIRO as a Marine National Facility (available to all research institutions by competitive application). The *Southern Surveyor* is near the end of its serviceable life and has had only a limited number of days at sea in recent years, but funding has now been allocated to increase its annual sea days and to build a replacement vessel that will operate for twice as many days per year. The planned replacement vessel will be a multipurpose research facility (not only for biodiversity research), but it will greatly increase Australia's capacity for research on the biota, especially offshore.

### Value, use, and impacts of marine biodiversity

Australia's marine biodiversity is the basis for many important commercial, tourism, and recreation activities. The overall value of ecosystem services from the world's oceans has been estimated to be about US$21 trillion per year [61], and the value of Australia's marine industry is around $38 billion, rising 42% since 2001 [62]. Although Australia's fisheries are more limited than those of some other countries, they nonetheless provide an important source of wealth and recreation in most coastal areas of Australia. The commercial fisheries are concentrated on high-value, but low-tonnage benthic species and products (Catch statistics or values are not detailed here). Australia is a federation of states and is governed at state and national levels; some fisheries (called Commonwealth fisheries) are managed by the Australian Government, through the Australian Fisheries Management Authority, whereas others (generally nearshore) are managed by states and territories. Data on the production and values of these fisheries are given in reports by the Australian Bureau of Agricultural Resource Economics (ABARE, http://www.abare.gov.au/). In outline, in 2006-7, Australia's commercial fisheries (including aquaculture) produced about 240,000 metric tons of seafood valued at about $2.18 billion [63]. This fell to $1.34 billion in 2007-8, continuing a trend since 2000-1 [65]. In addition, recreational and subsistence fisheries form an important part of Australia's coastally focused culture and make a major contribution to the Australian way of life. Recreational catches are thought, for some fish species, to be larger than the commercial catch, although it is difficult to obtain data [63]. Recreational fishing and marine tourism in general were estimated to contribute $11.6 billion to the Australian economy in 2006-7 [62].

Information on the status of Australian Commonwealth fisheries is available from the Australian Government Department of Agriculture, Fisheries and Forestry (http://www.daff.gov.au/fisheries) and in publications of the Bureau of Resource Sciences (BRS, http://www.daff.gov.au/brs/fisheries-marine/publications). There has been a disturbing trend toward increased overfishing in Commonwealth fisheries [64]. This trend is probably being arrested, however, at least for several major fisheries, as a result of firm, recent measures being taken following a directive to the Australian Fisheries Management Authority in November 2005 by the Australian Government Minister for Fisheries, Forestry and Conservation and the introduction of a Commonwealth Fisheries Harvest Strategy Policy. The picture looks more encouraging in the latest report [65], where assessment of stock status by Australian Biological Resources Study is combined for the first time with assessment of the economic status of fisheries by ABARE. Still a number of stocks are overfished or subject to overfishing, and a number have been classified as uncertain. In 2009 the UN Food and Agriculture Organization rated Australian prawn fisheries as the best managed fisheries in the world – the Northern Prawn Fishery is among the first major fisheries in the world to fully embrace both environmental sustainability and economic efficiency in an operational management system. The authors of a theme commentary for the 2006 State of Environment Report [66] found it difficult to obtain adequate data on the status of state and territory fisheries, but concluded that they varied in their status, from stable to overexploited. The problem of illegal, unreported, and unregulated fishing remains a significant issue, with uncertain impact, especially in the northern and sub-Antarctic waters of Australia [65].

### National policy on biodiversity

Australian and state governments have the conservation of biodiversity firmly embedded in their policies, the major instrument being the Australian Government's EPBC Act (1999, under revision). At present, Australia's Biodiversity Conservation Strategy is undergoing revision, and a draft has been distributed for consultation [67] (http://www.environment.gov.au/biodiversity/index.html.). The strategy lists a number of actions to be taken by all Australians, and identifies the main threats to biodiversity as follows:

- Climate change (resulting in conditions such as prolonged drought)
- Invasive species
- Loss, fragmentation, and degradation of habitat
- Unsustainable use of natural resources
- Changes to the aquatic environment and water flows
- Inappropriate fire regimes.

Naturally, its focus is largely terrestrial, but there is more emphasis on marine biodiversity than in the earlier edition of the strategy and there has been explicit attention given recently to marine biodiversity (e.g., [68]).

Australians value their marine biodiversity for its aesthetic value, apart from the range of commercial values. Marine tourism is a major industry, valued at about $11.6 billion in 2006-7 [62]. Australians also increasingly recognize the ecosystem services provided by healthy marine environments including those provided by marine biodiversity. Particularly under the threat of climate change and sea-level rise, coastal stabilization by mangroves and seagrasses is perhaps the best known of them. Currently, there are no good estimates of the value of ecosystem services, but this is an active research area.

### Major threats to biodiversity in the region and their relative importance over space and time

Threats to biodiversity are widely discussed [66] and include the usual list of pressures (pollution, exploitation, invasive species, effects of human activities, and habitat loss), varying, of course, in importance around the Australian coastline, depending on population densities and the locations of industries. The Marine Biodiversity Decline Working Group [68] lists the five key systemwide threats as climate change, resource use, land-based impacts, marine biosecurity, and marine pollution.

There has been substantial research in Australia into the effects of fishing – directly on target species, directly on nontarget species, and indirectly through impacts on habitats or through ecosystem connections [44], [69]–[76]. There has been work on the amelioration of these effects by direct means (e.g., turtle exclusion devices in prawn trawls; improved setting techniques on longlines [77]–[80]) and by spatial management. Spatial management includes not only the conventional measures such as permanent or seasonal closures and marine protected areas (MPAs) (e.g., in the south-east [47]; on the Great Barrier Reef [73], [81]–[83]; or in the sub-Antarctic, http://www.environment.gov.au/coasts/mpa/publications/pubs/heard-proposal.pdf), but also adjustments to the movements of fishing vessels in response to real-time data, so as to reduce bycatch of restricted species [84], [85]. Recent work has been investigating how to structure incentives so that it is in fishers' interests to avoid bycatch and offsets to compensate for any unavoidable mortality [86], [87].

There has also been research on invasive species [55], though rather less on the effects of invasive species on biodiversity [88]–[91]. There is a developing national system for prevention and management of marine pests that will manage all known international and national vectors [55], and Australia remains one of the few countries to have successfully eradicated a marine invader [92].

There is increasing evidence of changes in the ranges of, and in the interactions between, native marine species under the influence of climate change (e.g., [2], [90], [92]). These present not only important practical problems, but also fascinating studies in ecological interactions.

### Conservation areas

Australian and state governments are committed to a policy of establishing a National Representative System of Marine Protected Areas (NRSMPA) (see http://www.environment.gov.au/coasts/mpa/nrsmpa/). Australia has made major achievements in the establishment of protection for marine biodiversity and a number of them are world renowned (e.g., Ningaloo Marine Park, Macquarie Island Marine Reserve, Heard Island and McDonald Islands Marine Reserve, and of course the Great Barrier Reef Marine Park – http://www.gbrmpa.gov.au/).

As of 2004, the NRSMPA covered approximately 64.8 million hectares, or 7% of Australia's marine jurisdiction, excluding the Australian Antarctic Territory. At that date, there were MPAs of some kind in 41 of the 60 IMCRA mesoscale bioregions on the shelf. This still falls short of the stated goal of establishing at least 10% of each marine bioregion within MPAs by 2012, but Australian and state governments remain committed to reaching that goal and planning is active around Australia. The NRSMPA system expanded to include nearly 9.5% of Australia's marine waters in 2008. However, much of this increased area of MPAs is neither on the continental shelf, nor within the nearshore (generally 3 nautical miles) state waters, where much of the highly valued biodiversity and many of the pressures are located [47]. Many protected areas are designated for multiple use, and zonation and establishment of management plans lags behind the initial declaration of MPAs. The NRSMPA is underpinned by the principles of comprehensiveness, adequacy, and representativeness (CAR, http://www.environment.gov.au/coasts/mpa/nrsmpa/index.html), but there is uncertainty about whether this will be achieved in practice given the conflicting political and socioeconomic pressures.

To return to the positive, however, the policy toward a NRSMPA continues to be pursued actively. It is an established component of Australian biodiversity management, having endured through a decade and numerous changes of Australian and state governments.

### Endangered species or systems

Numbers of listed threatened species are indicated in the Results section above. All (so far) are vertebrates. This is not to say that no invertebrates, or members of other kingdoms, are at risk, but simply that they have not been identified and submitted for listing. It is also more difficult to confirm the status of marine species as threatened and have them listed, for a number of reasons, including lack of widespread community and scientific monitoring (compared with terrestrial environments), and lack of reliable baseline data. No whole systems have been identified as threatened, though some are under various pressures as indicated above, and the areas of most concern are near the human population centers, especially in the southeastern part of the continent, where rising temperature is also likely to be most pronounced.

### Potential and priorities for future discovery and research

With only one oceangoing research vessel, Australia lags behind many other countries in its ability to survey its large EEZ. Robots (Argo floats and gliders) have become increasingly useful in collecting physical information — more data have been collected in the last two years from the Argo floats than previously existed — and there are plans to develop a biological sampling capacity on these robots, although possibly at the microbial level. Relatively new technologies, like swath bathymetry, are providing the ability to rapidly map and visualize the bottom in deeper waters, and this is essential for targeting subsequent biodiversity sampling. Biodiversity is increasingly being investigated at the taxonomic and genetic level to determine not only where (or which) species occur, but also how the populations are structured in relation to the physical environment.

It is clear that Australia will never have the capacity to survey its entire EEZ and although high-priority areas will be targeted, many areas will remain unsurveyed or surveyed at low density. In response, Australia's Marine Biodiversity Hub is developing new statistical approaches to predicting biodiversity, using biological interpretations of physical data [57]. These techniques (which incorporate uncertainty) will support ongoing management and conservation of biodiversity while discovery continues. They offer the prospect, first, of having a surrogate-based map of predicted biota to tentatively fill the gaps in our present maps of the occurrence of species and, second, of operating an efficient, cost-effective survey program to progressively ground-truth such a tentative map with real discoveries. All recent voyages have shown that when we do that, a large number of new species will be added to Australia's inventory.

The object of modern biodiversity research is not, of course, merely to discover new species. There is increasing realization in Australia that for management of human activities, it is necessary to understand the ecosystem linkages all the way from the smallest organisms to the largest. Finding new species is of course an important component of that understanding, but the “voyages of discovery” of the future will also focus on processes.

This paper has stressed that we are particularly weak in our understanding of pelagic systems, especially the small organisms in those systems. Research efforts on phytoplankton have been small, but significant, for many years. Energetic early work on zooplankton was not followed up with substantial collection and monitoring efforts, but it is anticipated to increase with the recent establishment of new Continuous Plankton Recorder lines under Australia's Integrated Marine Observing System (http://imos.org.au/auscpr_intro.html). More significantly, our emphasis on microbial systems has been minuscule in the past, but is now accelerating in several major scientific institutions (CSIRO, AIMS, several universities) with the aid of modern genomic techniques. We predict that microbiological studies will be a major area of new discoveries in Australian marine biodiversity in the near future.

An incredible amount of work has been undertaken to describe Australia's marine biodiversity by agencies, museums, and dedicated individuals, but throughout this paper we have stressed that we have only scratched the surface of what is out there. To conserve Australia's exceptional marine biodiversity requires the ability to make good decisions now in the face of uncertainty. Australian scientists, most recently through the Marine Biodiversity Hub, are developing new methods to predict and map marine biodiversity using a biological interpretation of physical surrogates (e.g., [57]), and scientists in the CERF Applied Environmental Decision Analysis Hub are further developing what is already the world's most used conservation planning tool [94]. The Environment Protection and Biodiversity Conservation Act 1999 (EPBC Act) lists Commonwealth marine areas as one of seven matters of national environmental significance. The EPBC Act is being used to support comprehensive marine bioregional planning, including establishing a national representative system of marine protected areas by 2012 to meet Australia's commitments under the Convention on Biological Diversity and the 2002 World Summit on Sustainable Development. Yet, as we increasingly recognize the many irreversible changes in the marine environment through invasive species, global warming, and ocean acidification, it is clear that passive conservation alone will not be sufficient to protect our biodiversity. As Australia's Draft Biodiversity Conservation Strategy states, “business as usual is no longer an option.” In the face of the increasing loss and redistribution of species, conserving the ecological and social values of biodiversity will require an increasingly interventionist role.

## Supporting Information

Table S1 Estimated numbers of marine species for each higher taxon represented in Australian waters and an indication of the taxonomic expertise for those groups within the Australian study area. In the column headed Australian known marine species (est.), are counts of species either described in litt. or anticipated to be present in Australian waters on the basis of projected percentages of global totals but not yet recorded. A smaller but more reliable number, formally described and listed in four major electronic databases are shown in the column of that heading. The databases are: The Australian Faunal Directory, AFD (ABRS); Codes for Australian Aquatic Biota, CAAB (CSIRO); OBIS (maintained in Australia by CSIRO); and the Online Zoological Collections of Australian Museums, OZCAM. The column headed Australian unknown species (est.) gives expert opinions on species not yet recognised as new species, or undiscovered, i.e. never collected. Numbers of introduced species come from [55]. Numbers of taxonomists are from a survey by the ABRS in 2003; figures may include present and past practicing experts and numbers may be fewer now. The number of spp in AFD, CAAB, OBIS and OZCAM, expressed as a % of the Australian known marine species (est). is used in our estimate of State of Knowledge: 5 =  very well-known (>80% of est Aust species are in databases, ID guides <20 years old, and current taxonomic expertise); 4 =  well-known (>50% of est Aust species are in databases, ID guides <50 years old, some taxonomic expertise), 3 =  poorly known (<50% species of est Aust species are in databases, ID guides old or incomplete, no present expertise within region), 2 =  very poorly known (only few species in databases, no ID guides, no expertise), 1 =  unknown (no species in databases, no ID guides, no expertise). Spp  =  species. **** Table S1 is a protected Excel file ****
(0.07 MB XLS)

Table S2 Method for combining provincial bioregions from IMCRA 4.0 to approximate the LMD (Lyne et al. 2000). These are equivalent to LME (2002) and Spalding et al. 's (2007) Provinces for the Australian region. Records in the Australian Faunal Directory (AFD) are indexed by IMCRA 4.0 bioregions, not by the LMD which are more suitable for the present exercise. At the Provincial level, IMCRA 4.0 identifies Provinces, and Transition zones between them. For this article, records from those Provinces and Transition Zones were combined as follows to approximate the LMDs.
(0.05 MB DOC)

Table S3 Estimated numbers of described and undescribed species per taxon, by Large Marine Domain. Species richness estimates were based on AFD, but only those records with valid locations at LMD level were included. For many faunal groups represented in Table S1, there were insufficient data at LMD level.
(0.08 MB DOC)

Text S1 Identification guides and other guides to Australian biota. Bibliography 1 lists identification guides (by the definition used for this Census of Marine Life Regional Synthesis Collection). Bibliography 2 lists items that are not identification guides by Census of Marine Life definition, but important reference works on marine fauna and flora of Australian Region.
(0.11 MB DOC)
