# Supplementary material for: Marine Biodiversity in the Australian Region
Source: PLoS One. 2010 Aug 2;5(8):e11831. doi: 10.1371/journal.pone.0011831 (PMC2914019; doi:10.1371/journal.pone.0011831)
Supplement: Table S2 — Method for combining provincial bioregions from IMCRA 4.0 to approximate the LMD (Lyne et al. 2000). These are equivalent to LME (2002) and Spalding et al. 's (2007) Provinces for the Australian region. Records in the Australian Faunal Directory (AFD) are indexed by IMCRA 4.0 bioregions, not by the LMD which are more suitable for the present exercise. At the Provincial level, IMCRA 4.0 identifies Provinces, and Transition zones between them. For this article, records from those Provinces and Transition Zones were combined as follows to approximate the LMDs. (0.05 MB DOC) [file pone.0011831.s002.doc]

## Table S2. Method for combining provincial bioregions from IMCRA 4.0 to approximate the LMD (Lyne et al. 2000). These are equivalent to LME (2002) and Spalding et al.’s (2007) Provinces for the Australian region.

Records in the Australian Faunal Directory (AFD) are indexed by IMCRA 4.0 bioregions, not by the LMD which are more suitable for the present exercise. At the Provincial level, IMCRA 4.0 identifies Provinces, and Transition zones between them. For this article, records from those Provinces and Transition Zones were combined as follows to approximate the LMDs:

| **LME 2002** | **MEOW 2007 Province** | **Australian LMD name 2000** | **Contains these IMCRA 4.0 provinces** | **And these IMCRA 4.0 transition zones** | **Classified by IMCRA 4.0 as** |
| --- | --- | --- | --- | --- | --- |
|  |  |  |  |  |  |
| 40 | 33 & 35 | North Eastern | 20,18,40,17, | 41,19,16,15,39 | Tropical |
| 41 | 55 | Eastern Central | 38,12,13 |  | Warm temperate |
| 42 | 56 | South Eastern | 35,36,10 | 11,37,34,9 | Cold temperate |
| 43 | 57 | South Western | 33,8,31 | 32,7 | Warm temperate |
| 44 | 58 | Western Central | 6,29 | 30,28,5 | Subtropical |
| 45 | 34 | North Western | 4,27,2 | 3 | Tropical |
| 39 | 32 | Northern | 25 | 1,26 | Tropical |
|  |  |  |  |  |  |
| not covered | 36 | Norfolk (Lord Howe & Norfolk Is) | 14,21 |  | Warm temperate |
|  |  |  |  |  |  |
| not covered | 59 | Macquarie | 24 |  | Cold temperate |
|  |  |  |  |  |  |
| not covered | 59 | Kerguelen (HIMI) | not covered |  | Cold temperate |
|  |  |  |  |  |  |
| not covered | 27 | Sunda (Keeling-Cocos and Christmas) | 22,23 |  | Tropical |
