# Supplementary material for: Marine Biodiversity in the Australian Region
Source: PLoS One. 2010 Aug 2;5(8):e11831. doi: 10.1371/journal.pone.0011831 (PMC2914019; doi:10.1371/journal.pone.0011831)
Supplement: Table S3 — Estimated numbers of described and undescribed species per taxon, by Large Marine Domain. Species richness estimates were based on AFD, but only those records with valid locations at LMD level were included. For many faunal groups represented in Table S1, there were insufficient data at LMD level. (0.08 MB DOC) [file pone.0011831.s003.doc]

## Table S3. Estimated numbers of described and undescribed species per taxon, by Large Marine Domain. Species richness estimates were based on AFD, but only those records with valid locations at LMD level were included. For many faunal groups represented in Table S1, there were insufficient data at LMD level.

| **AFD data** |  |  |  |  |  |  |  |  |  |  |  |  |
| --- | --- | --- | --- | --- | --- | --- | --- | --- | --- | --- | --- | --- |
| IRMNG GROUP | LMD1 NORTH EASTERN | LMD2 EAST CENTRAL | LMD3 SOUTH EASTERN | LMD4 SOUTH WESTERN | LMD5 WEST CENTRAL | LMD6 NORTH WESTERN | LMD7 NORTH | LMD8 NORFOLK | LMD9 MACQUARIE | LMD10 KERGUELEN | LMD11 SUNDA | AUSTRALIA ALL |
|  |  |  |  |  |  |  |  |  |  |  |  |  |
| Mammalia | 34 | 38 | 48 | 50 | 39 | 33 | 21 | 0 | 1 | 1 | 0 | 54 |
| Aves | 25 | 24 | 26 | 24 | 23 | 21 | 18 | 7 | 1 | 1 | 2 | 35 |
| Reptilia | 27 | 12 | 11 | 9 | 12 | 32 | 34 | 0 | 0 | 0 | 0 | 38 |
| Pisces | 2617 | 1746 | 1784 | 1247 | 1513 | 2112 | 1551 | 484 | 0 | 0 | 2 | 4336 |
| Tunicata | 380 | 178 | 291 | 353 | 208 | 167 | 127 | 16 | 0 | 0 | 5 | 755 |
| Cephalochordata | 6 | 2 | 2 | 1 | 3 | 4 | 2 | 1 | 0 | 0 | 0 | 8 |
| Echinodermata | 592 | 423 | 435 | 330 | 369 | 575 | 378 | 1 | 1 | 0 | 0 | 1097 |
| Hemichordata | 6 | 2 | 3 | 3 | 1 | 1 | 0 | 1 | 0 | 0 | 0 | 9 |
| Platyhelminthes | 79 | 30 | 53 | 46 | 4 | 59 | 45 | 0 | 1 | 1 | 0 | 190 |
| Nematoda | 136 | 111 | 104 | 34 | 19 | 6 | 6 | 0 | 36 | 13 | 0 | 285 |
| Annelida | 533 | 617 | 647 | 464 | 392 | 399 | 155 | 12 | 0 | 0 | 0 | 1132 |
| Echiura | 5 | 7 | 10 | 9 | 2 | 1 | 0 | 0 | 0 | 0 | 1 | 14 |
| Sipuncula | 30 | 28 | 24 | 22 | 14 | 13 | 7 | 1 | 0 | 0 | 0 | 59 |
| Brachiopoda | 19 | 21 | 29 | 25 | 8 | 12 | 7 | 1 | 3 | 4 | 1 | 43 |
| Mollusca | 215 | 185 | 247 | 164 | 164 | 132 | 80 | 13 | 7 | 7 | 0 | 457 |
| Entoprocta | 7 | 3 | 7 | 4 | 0 | 1 | 1 | 0 | 0 | 0 | 0 | 16 |
| Porifera | 404 | 323 | 608 | 151 | 213 | 251 | 125 | 0 | 0 | 2 | 2 | 1142 |
| **TOTAL** | **5115** | **3750** | **4329** | **2936** | **2984** | **3819** | **2557** | **537** | **50** | **29** | **13** | **9670** |
