# Supplementary material for: Marine Biodiversity in the Australian Region
Source: PLoS One. 2010 Aug 2;5(8):e11831. doi: 10.1371/journal.pone.0011831 (PMC2914019; doi:10.1371/journal.pone.0011831)
Supplement: Text S1 — Identification guides and other guides to Australian biota. Bibliography 1 lists identification guides (by the definition used for this Census of Marine Life Regional Synthesis Collection). Bibliography 2 lists items that are not identification guides by Census of Marine Life definition, but important reference works on marine fauna and flora of Australian Region. (0.11 MB DOC) [file pone.0011831.s004.doc]

## Text S1

***Identification guides and other guides to Australian biota.***

## Bibliography 1

## Identification guides.

This list is not exhaustive, but represents items from the lead author’s shelves plus items found by a simple web search.

Allen G (2004) Marine fishes of tropical Australia and South-East Asia. A field guide for anglers and divers. Perth: Western Australian Museum. 292 p.

Allen G, Steene R (1994) Indo-Pacific coral reef field guide. Singapore: Tropical Reef Research. 378 p.

**Allen GR, Swainston R (1993)** The marine fishes of north**-**western Australia: a field guide for anglers and divers. Perth: Western Australian Museum.

**Bennett I (1992) Australian seashores: a guide to the formation, animal and plant life of Australia’s seashores. Pymble, NSW: Harper Collins. 266 p.**

**Burn R (1972) A guide to the Ascoglossa or sap-sucking sea slugs of Australia. *Aust Nat Hist* March 1972 pp. 174–178.**

**Carcasson RH (1977)** A field guide to the reef fishes of tropical Australia and the Indo-Pacific region. London: Collins.

**Carcasson RH (1977)** [A field guide to the coral reef fishes of the Indian and West Pacific Oceans*.* London: Collins.](http://librariesaustralia.nla.gov.au/apps/kss?action=Display&mode=fulldisplay&target=nbd&queryid=124&startPos=3)

Cobb G, Willan RC (2006) Undersea jewels: a colour guide to nudibranchs*.* Canberra: Australian Biological Resources Study.

**Colin PL, Arneson C (1995)** Tropical Pacific invertebrates: a field guide to the marine invertebrates occurring on tropical Pacific coral reefs, seagrass beds and mangroves. Beverly Hills, CA: Coral Reef Research Foundation.

Dakin WJ, Bennett I (1987) Australian seashores. A guide to the temperate shores for the beach-lover, the naturalist, the shore-fisherman and the student*.* (Fully revised edition of 1952 classic) North Ryde, NSW: Angus & Robertson. 411 p.

Daley RK, Stevens JD, Last PR, Yearsley GK (2002) Field guide to Australian sharks and rays. Hobart: CSIRO Marine Research and Fisheries Research and Development Corporation. 84 p.

Dartnall A (1980) Tasmanian echinoderms*.* Hobart: Fauna of Tasmania Committee, University of Tasmania.

Deas W, Deas J (2005) Coral reefs: nature’s wonders. Perth: Western Australian Museum.

Ditlev, H, (1980) A field-guide to the reef**-**building corals of the Indo**-**Pacific*.* Rotterdam: W Backhuys.

Duke N (2006) Australia’s mangroves – the authoritative guide to Australia’s mangrove plants. Brisbane: University of Queensland.

Edgar G (1997, 2000, 2008). Australian marine life: the plants and animals of temperate waters. Kew: Reed Books, 1997, 2000, 544 p. (Second Edition) Sydney: New Holland, 2008, 624 p.

Edgar GJ, Last PR, Wells MW (1982) Coastal fishes of Tasmania and Bass Strait. Hobart: Cat and Fiddle Press.

Fabricius K, Alderslade P (2001) Soft corals and sea fans. A comprehensive guide to the tropical shallow-water genera of the Central-West Pacific, the Indian Ocean and the Red Sea*.* Townsville: Australian Institute of Marine Science. 264 p.

Fautin DG, Allen GR (1997) Anemone fishes and their host sea anemones: a guide for aquarists and divers. Perth: Western Australian Museum.

Furlani D, Gales R, Pemberton D (2007) Otoliths of common Australian temperate fish: a photographic guide. Collingwood, Vic: CSIRO Publishing. 208 p.

Glasby CJ, Fauchald K (2003). POLiKEY. An information system for polychaete families and higher taxa. **ABRS Version 2: 5 June 2003, http://www.environment.gov.au/biodiversity/abrs/online-resources/polikey/index.html**

Gomon M, Bray D, Kuiter R, editors (2008). Fishes of Australia's southern coast. Sydney: Reed New Holland. 928 p.

**Grey DL, Dall W (1983)** A guide to the Australian penaeid prawns Darwin: Northern Territory Govt. Printing Office [for the Dept. of Primary Production of the Northern Territory].

Hale HM (1927-1929) The crustaceans of South Australia. Parts I & II. Reprinted 1976. Adelaide: Flora and Fauna Handbooks Committee; AB James, Government Printer.

**Huisman JM (2000) Marine plants of Australia. Nedlands: University of Western Australia Press. ix, 300 p.**

**Huisman JM, McCarthy PM (2006-2007) Algae of Australia. Vols 1–3. Canberra: ABRS; Collingwood: CSIRO Publishing.**

**Hutchins B, Thompson M (2001)** [The marine and estuarine fishes of south-western Australia: a field guide for anglers and divers*.*](http://librariesaustralia.nla.gov.au/apps/kss?action=Display&mode=fulldisplay&target=nbd&queryid=144&startPos=1)  Perth: Western Australian Museum.

**Hutchins B, Swainston R (2001) S**[ea fishes of southern Australia**:** complete field guide for anglers and divers.](http://librariesaustralia.nla.gov.au/apps/kss?action=Display&mode=fulldisplay&target=nbd&queryid=146&startPos=1) **Smithfield, NSW: Gary Allen Pty Ltd.**

**Jones DS, Morgan GJ (1994) A field guide to crustaceans of Australian waters. Chatswood, NSW: Reed. 216 p.**

Kott P (2005) Catalogue of Tunicata in Australian waters. PDF file available online. Canberra: Australian Biological Resources Study. 301 p. http://www.environment.gov.au/biodiversity/abrs/publications/electronic-books/tunicates.html

Kuiter RH (1993) Coastal fishes of South-Eastern Australia. Bathurst, NSW: Crawford House Press. 437 p.

Kuiter RH (1999) Guide to sea fishes of Australia*.* Frenchs Forest, NSW: New Holland.

Last PR, Scott EOG, Talbot FH (1983) Fishes of Tasmania. Hobart: Tasmanian Fisheries Development Authority.

Last PR, Stevens JD (2009) Sharks and rays of Australia. Second Edition. Collingwood, Victoria & Cambridge, Mass: CSIRO Publishing & Harvard University Press. ix, 644 p.

Leis JM, Carson-Ewart BM, (2000) [Larvae of Indo**-**Pacific coastal fishes: an identification guide to marine fish larvae*.*](http://librariesaustralia.nla.gov.au/apps/kss?action=Display&mode=fulldisplay&target=nbd&queryid=60&startPos=5)  Leiden & Boston: Brill.

**Lovelock C (1993)** [Field guide to the mangroves of Queensland*.*](http://librariesaustralia.nla.gov.au/apps/kss?action=Display&mode=fulldisplay&target=nbd&queryid=64&startPos=1)  Townsville: Australian Institute of Marine Science.

Lowry J, Watling L, Berggren M (2009). Crustacea.net. An information retrieval system for crustaceans of the world. <http://www.crustacea.net/index.htm>

Lu CC, Dunning M (1982) Identification guide to Australian arrow squid (family Ommastrephidae). Melbourne: Victorian Institute of Marine Sciences.

May JL, Maxwell JGH (1986) [Field guide to trawl fish from temperate waters of Australia.](http://librariesaustralia.nla.gov.au/apps/kss?action=Display&mode=fulldisplay&target=nbd&queryid=154&startPos=1) Hobart: CSIRO Division of Fisheries Research.

McAuley R, Newbound D, Ashworth R (2002) Field identification guide to Western Australian sharks and shark-like rays. Perth: Dept. of Fisheries, WA.

**Munday PI, Harold AS, Winterbottom R (1999) Guide to coral-dwelling gobies, genus *Gobiodon* (Gobiidae), from Papua New Guinea and the Great Barrier Reef. Revue française d’Aquariologie 26(1-2): 53–58.**

**Neira FJ, Miskiewicz AG, Trnski T (1998)** [Larvae of temperate Australian fishes: laboratory guide for larval fish identification*.*](http://librariesaustralia.nla.gov.au/apps/kss?action=Display&mode=fulldisplay&target=nbd&queryid=74&startPos=1) Nedlands, WA: University of Western Australia Press.

Newman L, Cannon L (2005) Fabulous flatworms: a guide to marine polyclads. Canberra: Australian Biological Resources Study/ CSIRO Publishing. ABRS Electronic Identification Series.

**Norman MD, Reid A (2000)** [A guide to squid, cuttlefish and octopuses of Australasia*.*](http://librariesaustralia.nla.gov.au/apps/kss?action=Display&mode=fulldisplay&target=nbd&queryid=80&startPos=2) Moorabbin, Vic: CSIRO Publishing.

O’Sullivan D (1985) A general guide to the metazoan zooplankton groups of the Southern Ocean. Kingston, Tas: Antarctic Division, Dept. of Science.

O’Sullivan D (1982) A guide to the pelagic polychaetes of the Southern Ocean and adjacent waters. Kingston: Antarctic Division, Dept. of Science and Technology.

**Phillips DAB, Handreck CP, Bock PE, Burn R, Smith BJ & Staples DA (1984) Coastal invertebrates of Victoria. An atlas of selected species. Melbourne: Marine Research Group of Victoria and Museum of Victoria.**

Poore, GCB (2004) Marine decapod Crustacea of Southern Australia. A guide to identification. (with chapter on Stomatopoda by Shane Ahyong). Collingwood, Vic:: CSIRO Publishing. ix, 574 p.

**Ride WDL (1980)** [A guide to the native mammals of Australia*.*](http://librariesaustralia.nla.gov.au/apps/kss?action=Display&mode=fulldisplay&target=nbd&queryid=162&startPos=1)  Melbourne: Oxford University Press.

Ritz D, Swadling K, Hosie G, Cazassus F (2003) Guide to the zooplankton of south eastern Australia. Fauna of Tasmania Handbook No 10. Hobart: Fauna of Tasmania Committee, University of Tasmania.

**Sainsbury KJ, Kailola PJ, Leyland GG (1985)** [Continental shelf fishes of Northern and North**-**western Australia: an illustrated guide*.*](http://librariesaustralia.nla.gov.au/apps/kss?action=Display&mode=fulldisplay&target=nbd&queryid=86&startPos=1) Canberra: Clouston & Hall and Peter Pownall Fisheries Information Service. 375 p.

Scott TD, Glover CJM, Southcott RV (several editions, 1962-1980) The marine and freshwater fishes of South Australia. Handbook of the Flora and Fauna of South Australia. Adelaide: Government Printer of South Australia. 392 p.

Scott FJ, Marchant HJ, editors (2005) Antarctic marine protists*.* Canberra: Australian Biological Resources Study/ Australian Antarctic Division. 563 p.

Shepherd SA, Thomas IM, editors (1982) Marine invertebrates of southern Australia. Part I. Adelaide: Flora and Fauna Handbooks Committee: DJ Woolman, South Australian Government Printer. 491 p.

Shepherd SA, Thomas IM, editors (1989) Marine invertebrates of southern Australia. Part II. Adelaide: Flora and Fauna Handbooks Committee: South Australian Government Printing Division. 900 p.

Shepherd SA, Davies M, editors (1997) Marine invertebrates of southern Australia. Part III. Adelaide: South Australian Research and Development Institute and Flora and Fauna Handbooks Committee. 1264 p.

Simpson K, Day N (2004) [Field guide to the birds of Australia. Seventh edition.](http://librariesaustralia.nla.gov.au/apps/kss?action=Display&mode=fulldisplay&target=nbd&queryid=6&startPos=2) Camberwell, Vic: Penguin/Viking. 382 p.

Simpson K, Day N (1999) Simpson & Day’s CD Birds of Australia 5.0 with 655 birdsongs. West Pennant Hills, NSW: Natural Learning

Slater P, Slater P, Slater R (2003) [The Slater field guide to Australian birds.](http://librariesaustralia.nla.gov.au/apps/kss?action=Display&mode=fulldisplay&target=nbd&queryid=8&startPos=3) Frenchs Forest, NSW: New Holland.

Smith BJ (1995) Tamar intertidal invertebrates: an atlas of the common species. Launceston: Queen Victoria Museum and Art Gallery.

**Swadling K, Slotwinski A, Ritz D, Gibson J, Hosie G (2008) Guide to the marine zooplankton of south eastern Australia.** Version 1.0 June 2008. **(**[www.tafi.org.au/zooplankton](http://www.tafi.org.au/zooplankton))

**Timms BV (2004)** [An identification guide to the fairy shrimps (Crustacea: Anostraca) of Australia*.*](http://librariesaustralia.nla.gov.au/apps/kss?action=Display&mode=fulldisplay&target=nbd&queryid=94&startPos=1)  Albury, NSW: CRC for Freshwater Ecology.

**Veron JEN (1993) Corals of Australia and the Indo-Pacific. 2nd Edition. Honolulu: University of Hawaii Press. xi, 644 p.**

Waycott M, McMahon K, Mellors J, Calladine A, Kleine D (2004) A guide to tropical seagrasses of the Indo-West Pacific. Townsville: James Cook University.

**Wells FE (1984)** [A guide to the common molluscs of south-western Australian estuaries / by Fred E. Wells; with photography by Clayton W. Bryce.](http://librariesaustralia.nla.gov.au/apps/kss?action=Display&mode=fulldisplay&target=nbd&queryid=98&startPos=1) Perth: Western Australian Museum.

Williams R, McEldowney A (1990) A guide to the fish otoliths from waters off the Australian Antarctic Territory, Heard and Macquarie Islands*.* Kingston, Tasmania: Antarctic Division, Dept. of the Arts, Sport, the Environment, Tourism and Territories.

Wilson B (1993) Australian marine shells. Prosobranch gastropods. Part 1. Kallaroo, WA: Odyssey Publishing. 408 p.

Wilson B (1994) Australian marine shells. Prosobranch gastropods. Part 2 (Neogastropods). Kallaroo, WA: Odyssey Publishing. 370 p.

Wilson RS, Hutchings PA, Glasby CJ, editors (2003) Polychaetes: an interactive identification guide. Melbourne: CSIRO Publishing.

Wilson SK, Swan G **(**2008) [A complete guide to reptiles of Australia.](http://librariesaustralia.nla.gov.au/apps/kss?action=Display&mode=fulldisplay&target=nbd&queryid=106&startPos=1)  Sydney: New Holland Publishers.

Womersley HBS (1984) The marine benthic flora of southern Australia. Part I. Adelaide: Flora and Fauna Handbooks Committee: DJ Woolman, South Australian Government Printer, Adelaide. 329 p.

Womersley HBS (1987) The marine benthic flora of southern Australia. Part II. Adelaide: Flora and Fauna Handbooks Committee: South Australian Government Printing Division. 484 p.

Womersley HBS (1994) The marine benthic flora of southern Australia . Rhodophyta. Part IIIA. Canberra: Australian Biological Resources Study. 508 p.

Womersley HBS (1996) The marine benthic flora of southern Australia. Rhodophyta. Part IIIB. Canberra: Australian Biological Resources Study. 392 p.

Womersley HBS (1998) The marine benthic flora of southern Australia. Rhodophyta. Part IIIC. Adelaide: State Herbarium of South Australia. 535 p.

Womersley HBS (2003) The marine benthic flora of southern Australia. Rhodophyta. Part IIID. Adelaide: Australian Biological Resources Study and State Herbarium of South Australia. 533 p.

## Bibliography 2

## Not Identification guides by Census of Marine Life definition, but important reference works on marine fauna and flora of Australian Region

#### Fauna of Australia

Beesley PL, Ross GJB, Wells A, editors (1998) Mollusca: the southern synthesis. Fauna of Australia. Vol. 5, Parts A & B. Melbourne: CSIRO Publishing. 1234 p.

Beesley PL, Ross GJB, Glasby CJ, editors (2000) Polychaetes & allies: the southern synthesis. Fauna of Australia. Vol. 4A Polychaeta, Myzostomida, Pogonophora, Echiura, Sipuncula. Melbourne: CSIRO Publishing. 465 p.

Dyne GR, Walton DW, editors (1987) General articles. Fauna of Australia. Vol. 1A. Canberra: Australian Government Publishing Service. 339 p.

Glasby CJ, Ross GJB, Beesley PL, editors (1993) Amphibia & Reptilia. Fauna of Australia. Vol. 2A. Canberra: Australian Government Publishing Service. 439 p.

Walton DW, Richardson BJ, editors (1989) Mammalia. Fauna of Australia. Vol. 1B. Canberra: Australian Government Publishing Service. pp. 401–1227.

#### Zoological Catalogue of Australia

Hoese DF, Bray DJ, Allen GR, Paxton J (2006) Fishes. In: Beesley PL, Wells A, editors Zoological Catalogue of Australia. Volume 35. Australia: ABRS & CSIRO Publishing. Part 1, pp. 1–756; Part 2, pp. 757–1450; Part 3, pp. 1451–2150.

Burdon-Jones C, Kott P, Richardson BJ (1998) Hemichordata, Tunicata, Cephalochordata. In: Wells A, Houston WWK, editors. Zoological Catalogue of Australia. Vol. 34. Australia: ABRS & CSIRO Publishing. 298 p.

Davie PJF (2002) Crustacea: Malacostraca: Phyllocarida, Hoplocarida, Eucarida (part 1). In: Wells A, Houston WWK, editors. Zoological Catalogue of Australia. Vol. 19.3A. Melbourne: ABRS & CSIRO Publishing. 551 p.

Davie PJF (2002) Crustacea: Malacostraca: Eucarida (part 2): Decapoda — Anomura, Brachyura. In: Wells A, Houston WWK, editors. Zoological Catalogue of Australia. Vol. 19.3B. Melbourne: ABRS & CSIRO Publishing. 641 p.

Hooper JNA, Wiedenmeyer F (1994) Porifera. In: Wells A, editor. Zoological Catalogue of Australia. Vol. 12. Melbourne: ABRS & CSIRO Publishing. 624 p.

Lowry JK, Stoddart HE (2003) Crustacea: Malacostraca: Peracarida: Amphipoda, Cumacea, Mysidacea. In: Beesley PL, Houston WWK, editors. Zoological Catalogue of Australia. Vol. 19.2B. Australia: ABRS & CSIRO Publishing. 531 p.

Paxton JR, Hoese DF, Allen GR, Hanley JE (1989) Pisces: Pteromyzontidae to Carangidae. In: Longmore R, Walton DW, editors. Zoological Catalogue of Australia. Vol. 7. Canberra: AGPS. 665 p.

Poore GCB (2002) Crustacea: Malacostraca: Syncarida, Peracarida: Isopoda, Tanaidacea, Mictacea, Thermosbaenacea, Spelaeogriphacca. In: Houston WWK, Beesley PL, editors. Zoological Catalogue of Australia. Vol. 19.2. Melbourne: ABRS & CSIRO Publishing. 434 p.

Rowe FWE, Gates J (1995) Echinodermata. In: Wells A, editor. Zoological Catalogue of Australia. Vol. 33. Melbourne: ABRS & CSIRO Publishing. 510 p.

Scheltema AM, Gowlett-Holmes K, Lamprell KL, Healy JM, Lu CC (2001) Mollusca: Aplacophora, Polyplacophora, Scaphopoda, Cephalopoda. In: Wells A, Houston WWK, editors. Zoological Catalogue of Australia. Vol. 17.2. Melbourne: ABRS & CSIRO Publishing. 353 p.

#### Other publications that provide guides to the literature

**McCarthy PM, Orchard AE, editors (2007) Algae of Australia: introduction. Canberra: ABRS; Melbourne: CSIRO Publishing. 744 p. (also includes chapter “A bibliography of Australian algae” by Huisman JM, Entwisle TJ, pp. 158–197).**
